# A Review of the High-Power All-Solid-State Single-Frequency Continuous-Wave Laser

**DOI:** 10.3390/mi12111426

**Published:** 2021-11-20

**Authors:** Weina Peng, Pixian Jin, Fengqin Li, Jing Su, Huadong Lu, Kunchi Peng

**Affiliations:** 1 State Key Laboratory of Quantum Optics and Quantum Optics Devices, Institute of Opto-Electronics, Shanxi University, Taiyuan 030006, China; 201912607006@email.sxu.edu.cn (W.P.); pxjin@sxu.edu.cn (P.J.); lfq@sxu.edu.cn (F.L.); jingsu@sxu.edu.cn (J.S.); kcpeng@sxu.edu.cn (K.P.); 2 Collaborative Innovation Center of Extreme Optics, Shanxi University, Taiyuan 030006, China

**Keywords:** nonlinear loss, all-solid-state laser, single-frequency operation, high-power, intensity noise suppression, wide-band tunable laser

## Abstract

High-power all-solid-state single-frequency continuous-wave (CW) lasers have been applied in basic research such as atomic physics, precision measurement, radar and laser guidance, as well as defense and military fields owing to their intrinsic advantages of high beam quality, low noise, narrow linewidth, and high coherence. With the rapid developments of sciences and technologies, the traditional single-frequency lasers cannot meet the development needs of emerging science and technology such as quantum technology, quantum measurement and quantum optics. After long-term efforts and technical research, a novel theory and technology was proposed and developed for improving the whole performance of high-power all-solid-state single-frequency CW lasers, which was implemented by actively introducing a nonlinear optical loss and controlling the stimulated emission rate (SER) in the laser resonator. As a result, the output power, power and frequency stabilities, tuning range and intensity noise of the single-frequency lasers were effectively enhanced.

## 1. Introduction

High-power all-solid-state single-frequency continuous-wave (CW) lasers with perfect beam quality, low-intensity noise and narrow linewidth have attracted growing attention in the fields of basic scientific research and military such as quantum information [1,2], atomic physics [3], precision measurement, ultra-fine spectroscopy, laser radar, laser guidance and so on. However, with the rapid developments of sciences and technologies, the traditional single-frequency lasers with low output power cannot meet the development requirements of many fields. For example, in order to construct a multi-component quantum entangled light source, a single-frequency CW green laser with the output power up to tens of watts as well as the low-intensity noise, higher long-term output power stability and better beam quality is required. In the detections of gravitational wave, the required output power of a single-frequency CW laser reaches even over 100 watts [4]. So far, the high-power single-frequency CW lasers have been developed with the fibers, slabs, thin disks and cubic crystals and so on. The disk lasers [5,6] benefit from the relatively large cooling surface, small heat dissipation volume of the gain medium, and have better heat dissipation characteristics. They are widely used in the preparation of high-power continuous laser and high-energy pulsed laser. Due to the short absorption optical path of the disk gain medium, it is necessary to use large double specific mirrors to reflect the pump light multiple times to improve the pump absorption efficiency. It is relatively rare in the practical applications of the fine measurement fields that require high indicators such as laser output power stability and noise characteristics, as well as high technical requirements for ease of operation and maintenance in the later period. The relatively large cooling surface and small axial heat dissipation volume of the gain medium in the slab laser [7,8] are relatively easy to achieve a better heat dissipation. Slab lasers are easy to inject higher pump power to achieve high-power laser output. However, the complicated multiple transmission paths of the seed beam in the slab laser can easily lead to large differences in the X and Y axes of the amplified laser beam. In the field of fine cutting-edge research, the laser beam is required to have a high-purity Gaussian beam distribution. Even if the beam of the slab laser is shaped by the beam shaping system, it is difficult to meet this requirement. The single-frequency fiber laser [9,10,11,12,13,14] is limited by the laser damage threshold of the mode-selecting element and the power of the single-mode laser-diode, and the maximum output power is below the watt level. The optical fiber structure has relatively large heat dissipation surface and small heat dissipation volume, so that it has a better heat dissipation; at the same time, seed light employing master oscillator power amplification (MOPA) is transmitted in the optical fiber at the waveguide way, which is easy to maintain a high beam quality after amplification. The output power of the fiber MOPA is mainly affected by the nonlinear effect of the fiber laser. For scaling up the output power of a single-frequency CW laser, it is first necessary to improve the incident pump power. However, different from the disk lasers, slab lasers, and fiber lasers, the severe thermal effect in the solid-state lasers [15,16,17,18] has to be considered with the increase of the incident pump power because it can directly limit the increase of laser output power. Simultaneously, the laser mode competition is intensified with the increase of laser gain, which seriously damages the single-frequency characteristics, frequency stability and intensity noise characteristics of the laser. In order to overcome the difficulty, the laser amplifications including MOPA and injection-locked amplification can pave a good way to scale up the power and simultaneously keep the good single-frequency characteristic of a single-frequency laser. In 2005, M. Frede et al. introduced two sets of injection-locked amplification devices in the front and rear of the laser amplification system to amplify the low noise 2 W NPRO seed source laser to 12 W. Adopting this power amplification technique, the laser frequency can be simultaneously and precisely locked to the oscillation frequency of the high-power resonator. They eventually realized 195 W single-frequency CW 1064 nm laser output [16]. In 2012, in order to meet the demand of gravitational wave detection, Laser Zentrum Hannover adopted a single plane ring cavity as the seed source and realized a single-frequency CW laser with maximum output power of 220 W [18] by means of the combination of a traveling wave amplification and an injection-locked amplification. Though the laser amplification has demonstrated their powerful ability, the inevitable complexity and being sensitive to the variation of the environment further restrict their applications in many fields. Therefore, it is expected to directly scale up the output power of a single-frequency CW laser in a single resonator. In particular, Martin et al. developed a novel method to attain the single-longitudinal-mode (SLM) operation of the laser in 1997, which was achieved by introducing a nonlinear loss into a ring laser resonator [19]. The introduced nonlinear loss could increase the loss difference between the lasing and nonlasing modes. As a result, the oscillation of nonlasing mode was effectively suppressed, and a stable single-frequency CW laser was obtained. In 2005, Greenstein and Rosenbluh analyzed the influence of nonlinear spectral bandwidth on the SLM intracavity second-harmonic generation (SHG) [20]. Both studies can supply a good and effective way to develop high-power single-frequency CW laser by means of the nonlinear loss. On this basis, the physical condition of SLM operation based on the nonlinear loss was proposed. At the same time, depending on the nonlinear loss, the output power of the single-frequency laser was scaled, the power and frequency stability was improved, the continuous tuning range was expanded, and the intensity noise was suppressed.

## 2. High-Power All-Solid-State Single-Frequency CW 1064 nm Lasers

### 2.1. Physical Conditions of SLM Operation

**Figure 1:**
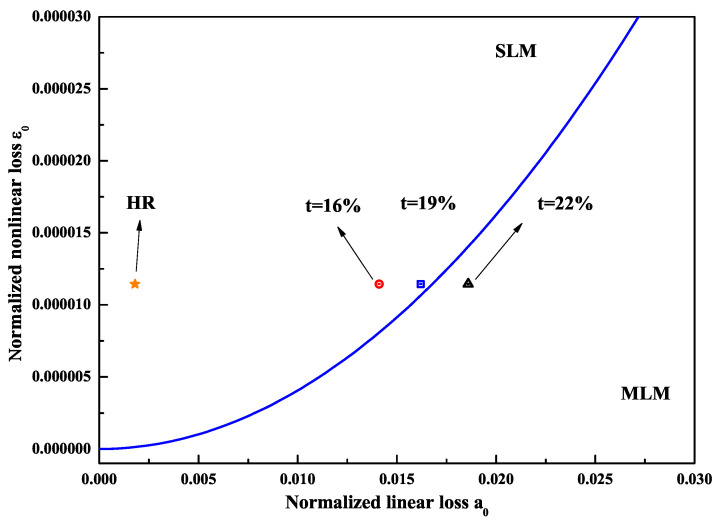
Dependence of nonlinear loss $\varepsilon_{0}$ on linear loss $\alpha_{0}$ for the criterion condition of SLM operation [21]. Reprinted with permission from [21] © The Optical Society.

When the incident pump power of the all-solid-state single-frequency CW laser is increased to scale up the output power, the mode competition becomes so intense that the multi-longitudinal mode (MLM) and mode hopping are easily occurred and observed. In order to suppress the oscillation of other side modes and maintain stable single-frequency operation of a laser, the traditional method adopted in the experiment was employing an intracavity etalon with suitable bandwidth. And the transmitted peak had to be locked to the oscillating frequency of the laser. In 1997, Martin, Clarkson and Hanna first presented that the axial mode hopping was suppressed by intracavity SHG since the lasing mode had half the nonlinear loss of the nonlasing mode. Later, Greenstein and Rosenbluh studied the influence of nonlinear spectral bandwidth on SLM intracavity SHG. On this basis, the physical conditions of SLM operation for high-power all-solid-state lasers was proposed [21].

(1) $$\frac{1}{2}-2{\mathrm{sinc}}^{2}\left(\frac{1.39}{2\gamma }\right)<\frac{\alpha_{0}}{\sqrt{{\left(\alpha_{0}-\varepsilon_{0}\right)}^{2}+4\varepsilon_{0}}-\left(\alpha_{0}+\varepsilon_{0}\right)}$$

where $\alpha_{0}$ and $\varepsilon_{0}$ were the normalized linear and nonlinear loss, respectively. γ was the ratio between the nonlinear spectral bandwidth of the nonlinear crystal and the gain bandwidth of the laser crystal. When the left side of Equation (1) was smaller than its right side, the laser would operate at the SLM state. The solid curve in Figure 1 showed the dependence between $\varepsilon_{0}$ and $\alpha_{0}$, which was obtained from Equation (1) when its left side equaled its right side. The region above the solid curve represented the left side of Equation (1) smaller than the right side, which resulted in SLM operation of the laser. On the contrary, the left side of Equation (1) was larger than the right side and thus the laser would operate at the MLM state. When the configuration of the resonator and the laser medium had been chosen, the parameter γ was a fixed value. In this case, we could adjust the parameters $\varepsilon_{0}$ or $\alpha_{0}$ to make the laser operate at the SLM state.

**Figure 2:**
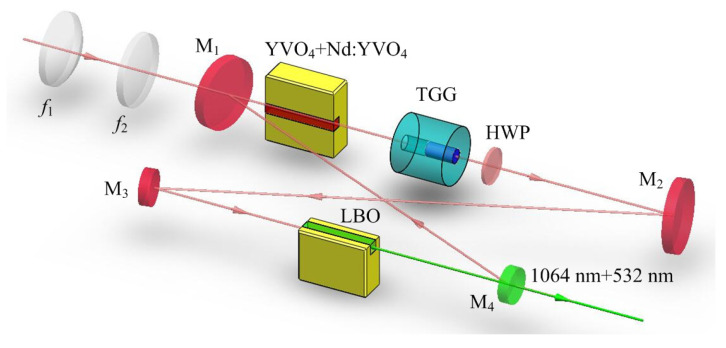
Schematic of the single-end laser-diode-pumped laser [21]. Reprinted with permission from [21] © The Optical Society.

Based on the presented physical conditions of the SLM operation, a homemade all-solid-state single-frequency 1064 nm laser was designed and built as shown in Figure 2. A bow-tie ring resonator cavity consisting of a telescope pump-coupled system, a gain medium, an optical diode, a nonlinear crystal and four cavity mirrors was constructed. The pump source was fiber-coupled laser-diode with the center wavelength of 888 nm. The pump radiation was imaged in the center of the laser gain medium. The laser gain medium was an a-cut composite YVO${}_{4}$/Nd:YVO${}_{4}$ rod, which had an undoped end cap of 3 mm and a 0.8% Nd-doped rod of 20 mm with a wedge angle of 1.5${}^{\circ }$ at the second end face. The front end face of the laser gain crystal was coated with anti-reflection (AR) films at 1064 and 888 nm, and the second end face was coated with AR films at 1064 nm. To maintain the unidirectional operation of the laser, an optical diode consisting of an 8 mm long terbium gallium garnet (TGG) rod surrounded by a magnetic field and an AR-coated half-wave plate at 1064 nm was applied. In order to effectively suppress the nonlasing mode oscillation of the laser, a type-I noncritical phase-matching lithium triborate (LBO) crystal with the dimensions of 3 mm × 3 mm × 18 mm was employed to introduce nonlinear loss into the laser resonator. The input coupler M${}_{1}$ and M${}_{2}$ were two convex mirrors with curvature radius of *R* = 1500 mm, where M${}_{1}$ was coated with high-reflective (HR) films at 1064 nm and AR films at 888 nm, and the M${}_{2}$ was coated with HR films at 1064 nm. The cavity mirror M${}_{3}$ and M${}_{4}$ were two plano-concave mirrors with curvature radius of *R* = −100 mm, where M${}_{3}$ was coated with HR films at 1064 nm, and the output coupler M${}_{4}$ was coated with fractional transmission at 1064 nm. According to the parameters of the designed laser resonator and Equation (1), the relationship between the normalized linear and nonlinear losses was theoretical predicted and the critical condition for SLM and MLM operation of the solid-state laser resonator was obtained.

**Figure 3:**
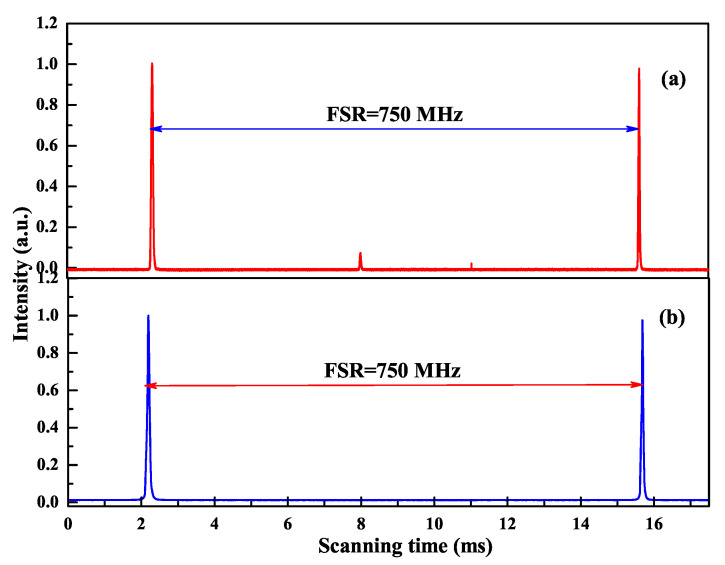
Longitudinal-mode structure of the laser (**a**) without and (**b**) with the nonlinear crystal LBO [21]. Reprinted with permission from [21] © The Optical Society.

In the experiment, the intracavity nonlinear loss was changed by manipulating the intracavity fundamental-wave laser intensity, which was implemented by changing the transmission of the output coupler. The larger the transmission of the output coupler, the smaller the intracavity fundamental-wave laser intensity and the nonlinear loss. It was clear that the nonlinear loss was large enough to suppress the oscillation of nonlasing modes and the mode hopping when the transmission of the output coupler was lower than 19% and the designed laser could well work with long-term stable SLM operation. Once the transmission of 22% was chosen, the nonlinear loss was too small to suppress the oscillation of the nonlasing modes and the MLM oscillation began and was observed, as shown in Figure 3. Finally, a stable SLM CW 1064 nm laser with the output power of 33.7 W was realized when the incident pump power and the transmission of the output coupler were 75 W and 19%, respectively. Because of the nonlinear loss, a 532 nm laser beam with 1.13 W was simultaneously produced. The optical-to-optical efficiency from 888 to 1064 nm conversion was better than 44.9% (considering the output power of 532 nm, the total efficiency was 46.5%). The measured long-term power stability was better than ±0.31% (peak-to-peak) for 7 h without mode hopping. The beam quality of the laser was measured by a M${}^{2}$ meter (M${}^{2}$-200, Spiricon Inc., Logan, UT, USA), and the values of M${}_{x}^{2}$ and M${}_{y}^{2}$ were 1.14 and 1.13, respectively. In the SLM range, the output powers of fundamental-wave and second-harmonic-wave lasers can be manipulated by changing the transmission of the output coupler [22]. The above experimental results have proven the experimental designs and the theoretical calculation are in good agreement. The proposed physical conditions provide a feasible way to design and develop the high-power all-solid-state single-frequency CW lasers with different wavelengths.

### 2.2. Employing Nonlinear Loss to Accurately Measure the Intracavity Loss of the Laser

**Figure 4:**
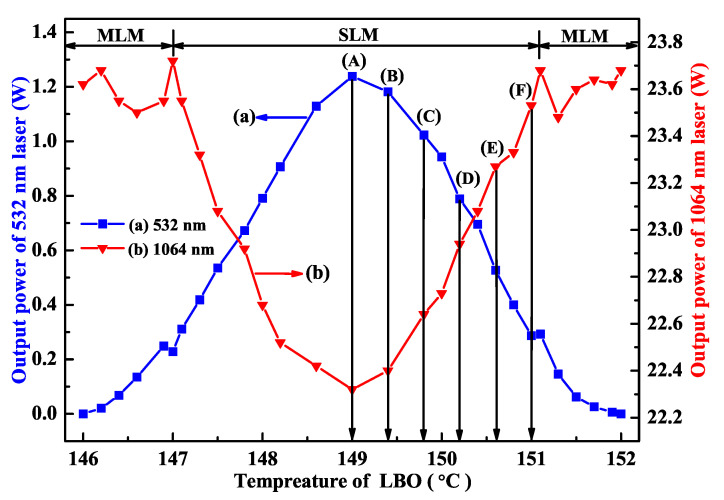
The output powers of the fundamental wave and second harmonic wave, respectively [23]. Reprinted with permission from [23] © Chinese Optics Letters.

After deliberately introducing the nonlinear loss to the laser resonator to realize the SLM operation of a single-frequency laser, a dual-wavelength single-frequency laser was quickly achieved. In this case, the output powers of the fundamental-wave and second-harmonic-wave lasers were decided by the intracavity linear and nonlinear losses when the incident pump power was ensured. In other words, the intracavity linear loss could be precisely measured by detecting the powers of the fundamental-wave and second-wave lasers. The relationship between them was described as follows [23],

(2) $$L=\frac{\eta I_{0}KP_{in}P_{f}^{2}-{\left(tP_{sh}\right)}^{2}-tP_{f}P_{sh}\left(t+\eta I_{0}\right)-\eta I_{0}tP_{f}^{2}}{\eta I_{0}P_{f}^{2}+tP_{sh}P_{f}}$$

where *L* was the intracavity round-trip loss, η was the nonlinear conversion factor, *I*${}_{0}$ was the saturation intensity, *K* was the pump factor, *P*${}_{in}$ was the pump power, and *t* was the transmissivity of the output coupler. *P${}_{f}$* and *P${}_{sh}$* were the output powers of the fundamental wave and second harmonic wave, respectively. In the measurement process, both powers of fundamental-wave and second-harmonic-wave lasers were changed by scanning the phase-matching temperature of the adopted frequency-doubling LBO crystal, which was depicted in Figure 4. According to the measured results and Equation (2), the measured linear loss of the designed single-frequency laser was 4.84%, and the standard deviation of the intracavity linear loss was 0.26%. Compared to traditional measurement technologies, the presented method can accurately measure the intracavity loss of laser resonator without replacing any optical elements, which reveals the simplicity and convenience as well as high precision of the presented method.

**Figure 5:**
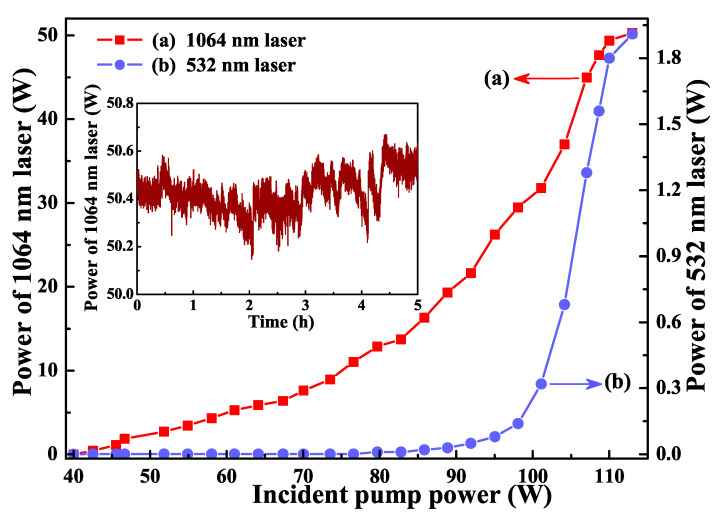
Output power of 1064 nm laser (**a**) and 532 nm laser (**b**) versus incident pump power. Inset showed power stability during 5 h [24]. Reprinted with permission from [24] © The Optical Society.

Depending on the measured intracavity linear loss, the incident pump power of the designed single-frequency laser increased to 113 W, the transmission of the output coupler was optimized to 25%, and an all-solid-state single-frequency CW 1064 nm laser was achieved [24] with a maximum output power up to 50.3 W. Simultaneously, a single-frequency 532 nm laser with the maximal output power of 1.91 W was generated because the intracavity LBO crystal was working at the optimal phase-matching temperature of 149.0 ${}^{\circ }$C. The output powers as a function of incident pump power were recorded in Figure 5. The corresponding optical-optical conversion efficiency was up to 46.20%. The beam quality M${}^{2}$ was measured by a M${}^{2}$ meter (M2SET-VIS, Thorlabs) and the measured values of M${}^{2}$${}_{x}$ and M${}^{2}$${}_{y}$ were 1.08 and 1.10, respectively. The measured degree of polarization of 1064 nm laser at the maximum output power was more than 110:1. The long-term power stability of 1064 nm laser was measured (Figure 5 inset) and the peak-to-peak power fluctuation was less than ±0.50% during 5 h. The measured spectral linewidth of 1064 nm laser was 206 kHz.

### 2.3. 101 W Single-Frequency CW 1064 nm Laser

**Figure 6:**
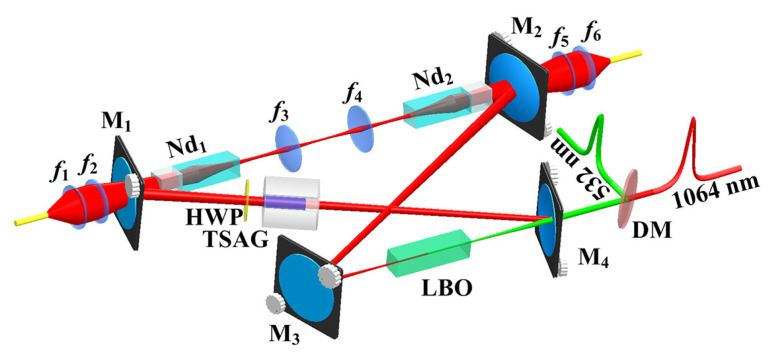
Schematic diagram of a 101 W single-frequency CW 1064 nm laser [25]. Reprinted with permission from [25] © The Optical Society.

**Figure 7:**
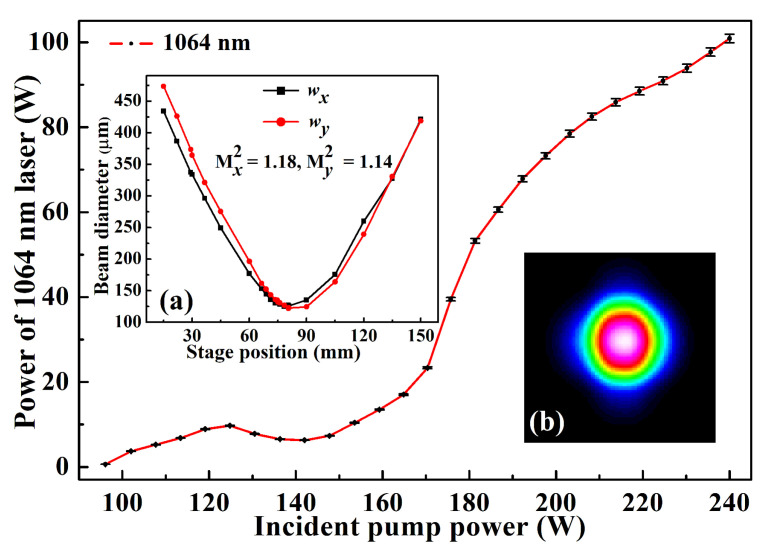
Output power of a 1064 nm laser versus the incident pump power. The insets were (**a**) the beam quality factor and (**b**) the profile of a 1064 nm laser at the maximum output power [25]. Reprinted with permission from [25] © The Optical Society.

To further scale up the power of a single-frequency laser, two laser-diode end-pumped identical Nd:YVO${}_{4}$ laser crystals were inserted in a single resonator to effectively alleviate the severe thermal load of the single laser crystal under high pump power. The diagram of the designed laser resonator was illustrated in Figure 6, where an imaging system was employed to realize cavity mode self-reproduction at the positions of both laser crystals. When the total incident pump power increased to 240 W, the output power of the single-frequency CW 1064 nm laser reached up to 101 W [25]. The variation of the output power with the increase of the incident pump power was depicted in Figure 7. Accompanying the single-frequency CW 1064 nm laser, there was about 1.91 W 532 nm laser leaked from the resonator. The overall optical-to-optical conversion efficiency achieved as high as 42.3%. At the maximal output power of the laser, the high-power stability was better than ±0.73%. The measured values of M${}^{2}$${}_{x}$ and M${}^{2}$${}_{y}$ were 1.18 and 1.14, respectively. The obtained caustic curve and the corresponding spatial beam profile were shown as insets (a) and (b) in Figure 7. Owing to the effect of the nonlinear loss induced by the nonlinear LBO crystal, the achieved CW laser can work with stable SLM operation. To the best of our knowledge, it is the highest output power of all-solid-state single-frequency CW 1064 nm laser in a single resonator without any amplifiers, so far. The obtained SLM laser with superior characteristics can well satisfy the requirements of quantum net, precise measurements and so on. The presented design scheme can also open a new way for further scaling up the output power of the all-solid-state single-frequency CW lasers.

## 3. High-Power All-Solid-State Single-Frequency and Frequency-Doubling CW Lasers

### 3.1. Thermal Lens Effect of the Magneto-Optical Crystal

**Figure 8:**
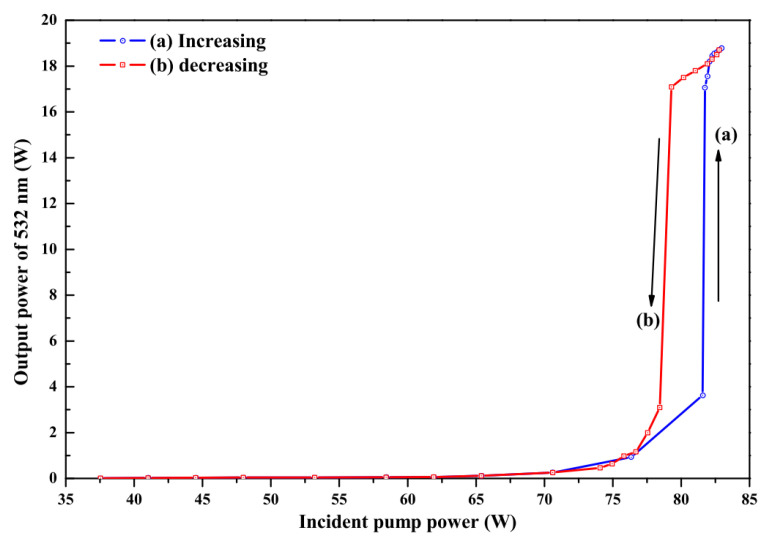
Output power of the 532 nm laser in the cases of (**a**) increasing and (**b**) decreasing the incident pump power with the bistability-like phenomenon [26]. Reprinted with permission from [26] © The Optical Society.

**Figure 9:**
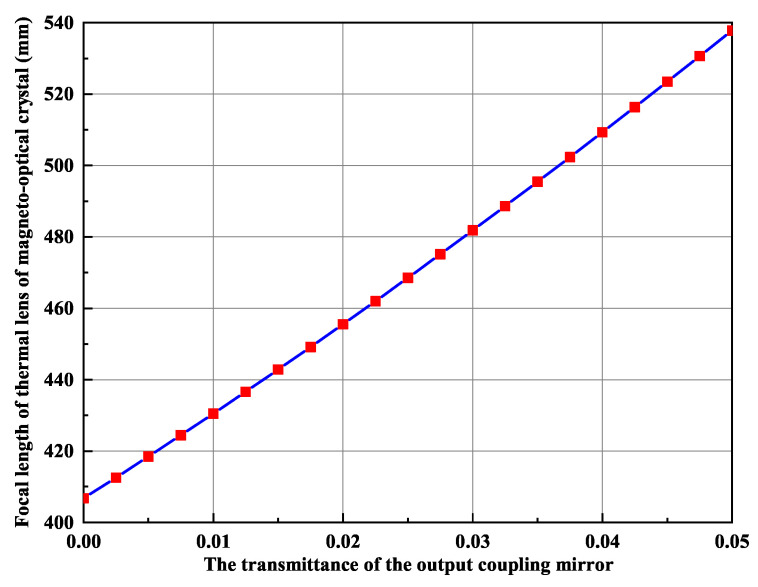
Focal length of thermal lens of magneto-optical crystal.

**Figure 10:**
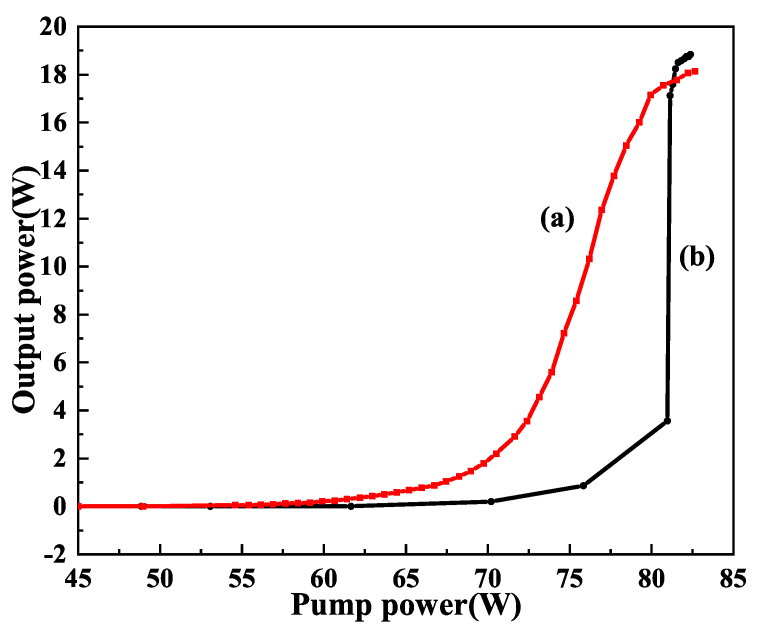
Output power of the 532 nm laser in the cases of increasing and decreasing the incident pump power without the bistability-like phenomenon. (**a**) The coupling mirror coated with the transmittance of 2% at fundamental-wave laser and (**b**) the coupling mirror coated with HR films at fundamental-wave laser.

It is well known that the intracavity intensity of the fundamental-wave laser should be as high as possible if we want to develop a high-power single-frequency CW 532 nm laser by means of intracavity SHG. Therefore, the output coupler is often coated with HR films at the fundamental wave and high transmission (HT) films at the second harmonic wave. However, due to the limited conversion coefficient of the nonlinear crystal, the residual fundamental-wave laser in the resonator because of the incomplete conversion can be absorbed by the magneto-optical TGG crystal acted as the optical diode. As a result, the TGG crystal also produced a serious thermal lens effect. It could be seen that in Figure 8 the serious thermal lens effect made the laser appearing bistability-like phenomenon and severely restricted the increase of the laser output power, even extremely damaged optical element. In order to reduce the influence of the thermal lens effect of the TGG crystal on the output characteristic of the laser, a method to control the intracavity intensity of the fundamental-wave laser was proposed [26], which was implemented by adopting an output coupler with a suitable transmission for the fundamental-wave laser. In the experiment, when the pump power was 80 W, we designed an output coupling mirror coated with a certain transmittance of 2% at fundamental-wave laser to adjust the intracavity intensity of the fundamental-wave laser. The intensity of the fundamental wave in the resonator was reduced from 567 W/mm${}^{2}$ to 505 W/mm${}^{2}$, and the thermal lens focal length of the TGG crystal increased from 408 mm to 458 mm, as shown in Figure 9. Evidently, the thermal lens effect of the TGG crystal had been effectively reduced. In particular, the bistability-like phenomenon of the laser was eliminated. Additionally, experimental results are shown in Figure 10. It can be seen that the output power of the SHG at 532 nm do not decrease due to the increase in the transmittance of the output coupling mirror and the operation stability of the laser has been improved.

### 3.2. Employing the Crystal with a Negative Thermo-Optic Coefficient to Actively and Dynamically Compensate for the Positive Thermal Lens Effect of the Magneto-Optical Crystal in Real Time

**Figure 11:**
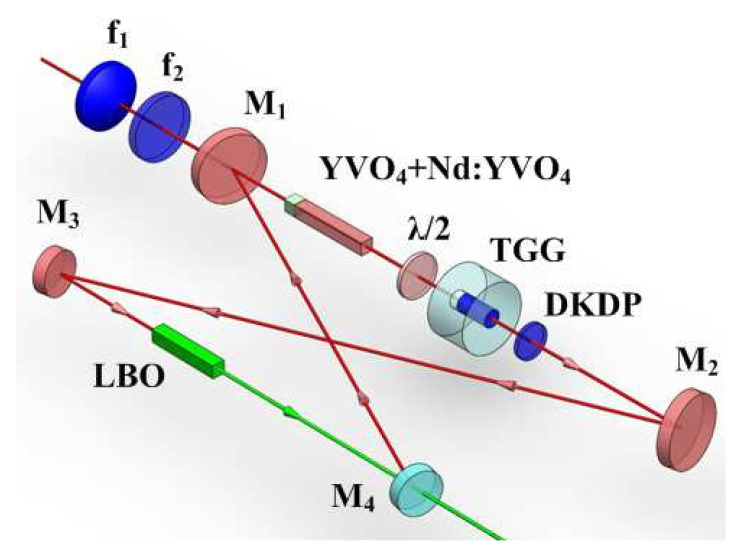
Experimental setup of active compensation for the thermal lens effect of the TGG crystal [27]. Reprinted with permission from [27] © The Optical Society.

**Figure 12:**
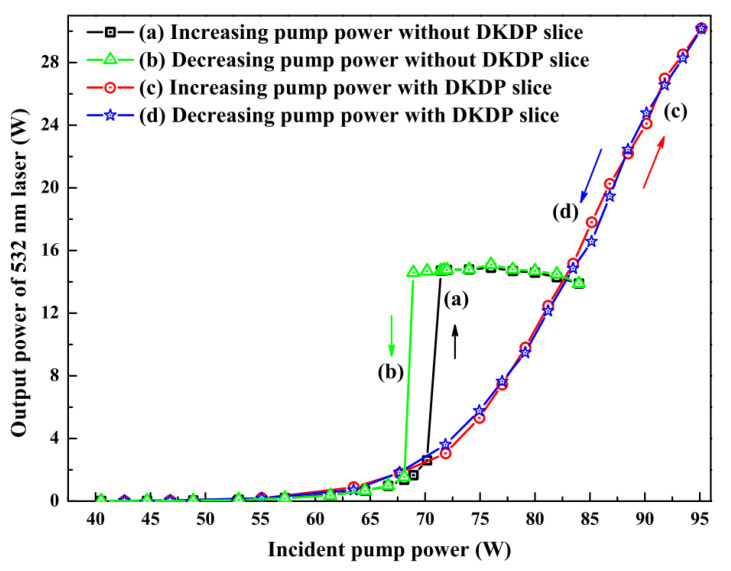
Output power of 532 nm laser versus pump power before and after the 1.6-mm-thick DKDP slice was inserted into the laser resonator [27]. Reprinted with permission from [27] © The Optical Society.

An effective scheme for improving the output power of a single-frequency CW laser was further developed by actively compensating the thermal lens effect of the TGG crystal [27]. In the experiment, a potassium dideuterium phosphate (DKDP) slice with negative thermo-optical coefficient was employed to actively compensate for the thermal lens in the TGG crystal. Due to the negative thermo-optical coefficient, the negative thermal lens effect would produce in DKDP crystal, which could actively and dynamically compensate for the positive thermal lens effect of the magneto-optical crystal in real time and eliminate the influence of the thermal lens of the magneto-optical crystal on the SHG output power. The optical resonator structure with DKDP crystal inserted into the cavity was as shown in Figure 11. Based on the precise measurement of the thermal focal length of the magneto-optical crystal, the thickness of the DKDP crystal was optimized to 1.6-mm-thick. The output power characteristic curves after compensation were shown in Figure 12. It demonstrated that the thermal induced stress saltation in the cavity element had been mitigated to a great extent and the bistability phenomenon of the laser output had been eliminated, which was helpful for prolonging the service life of the laser. Therefore, when the incident pump power was 95 W (with 82 W absorbed), a stable single-frequency 532 nm laser of 30.2 W was obtained, corresponding to an optical-to-optical conversion efficiency of 36.8%, with long-term stability of the output power better than ±0.5% for 3 h and the beam quality of the laser was measured by a M${}^{2}$ meter (M2SET-VIS, Thorlabs) to be 1.02 and 1.04 in the x and y planes, respectively. The longitudinal-mode structure of the laser was monitored by a Fabry–Perot interferometer with a free spectrum range (FSR) of 750 MHz and finesse of 250, which showed that the laser could operate in SLM stably [28]. So far, an all-solid-state single-frequency CW intracavity SHG at 532 nm have been obtained with the highest output power. Furthermore, it could be found that the laser was not saturated at injected pump power of 95 W. If more powerful pump source was provided, the output power could be further promoted.

## 4. Improvement of the Power and Frequency Stabilities

**Figure 13:**
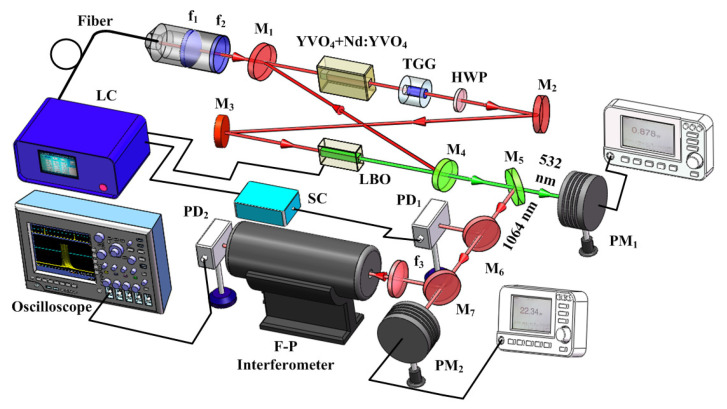
Experimental setup of the single-frequency laser with power stabilization system for fundamental wave [29]. Reprinted with permission from [29] © The Optical Society.

**Figure 14:**
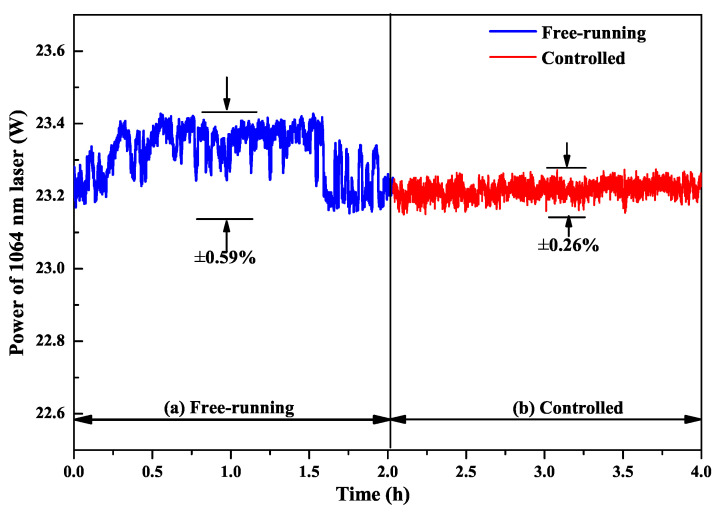
Power stability of 1064 nm laser in 4 h with two phases: (**a**) free-running phase and (**b**) controlled phase [29]. Reprinted with permission from [29] © The Optical Society.

**Figure 15:**
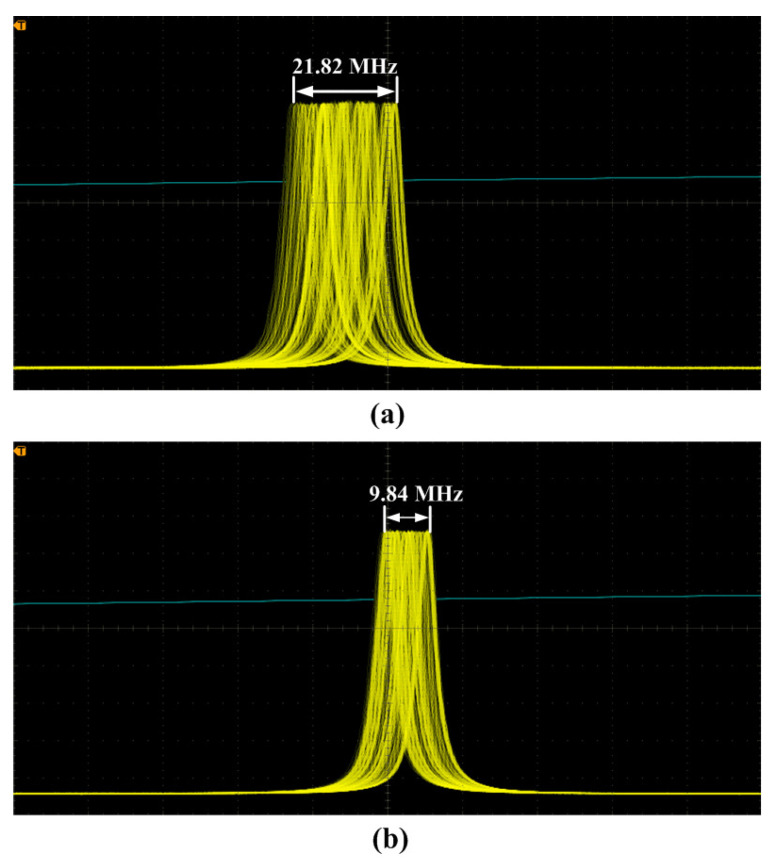
Frequency drift of 1064 nm laser in 1 min at two phases: (**a**) free-running phase and (**b**) controlled phase [29]. Reprinted with permission from [29] © The Optical Society.

From Section 2, it is well known that the output powers of the fundamental-wave and second-harmonic-wave lasers are directly decided by the intracavity nonlinear loss. Therefore, it is expected that the power and frequency stabilities of high-power all-solid-state single-frequency CW 1064 nm laser can be improved by feedback controlling the intracavity nonlinear loss [29]. To this end, the relationship between the output powers of the fundamental-wave and second-harmonic-wave lasers as well as the nonlinear loss must be first recorded. In the experiment, the manipulation of the intracavity nonlinear loss was achieved by direct controlling the phase-matching temperature of the nonlinear LBO crystal. The measured results were the same as Figure 4. Then, a suitable lock point in the laser system was optimized carefully to achieve the stabilization effect. This suitable stable lock point needs to meet the following conditions: (a) The laser should always operate in SLM state; (b) The output power of the laser should change monotonously with the temperature of the nonlinear LBO crystal; (c) The output power of the laser should change as quickly as possible with the temperature of the nonlinear LBO crystal. In order to select a suitable stable lock point, the operating temperature of the nonlinear LBO crystal should be limited to ±2 ${}^{\circ }$C around the best phase-matching temperature according to the physical conditions of single-frequency operation of high-power lasers. For this locking point, the output power of the laser should respond monotonously and quickly to the variation of the temperature of the nonlinear crystal in the range of the SLM operation. Lastly, two locking points around the optimal phase-matching temperature were available, where the one was 147.9 ${}^{\circ }$C, and the other was 150.4 ${}^{\circ }$C. The experimental setup of the single-frequency laser with feedback controlling system was depicted in Figure 13. Laser power and frequency stability before and after feedback control of nonlinear loss are shown in Figure 14 and Figure 15. The experimental results showed that the power fluctuation of the fundamental-wave 1064 nm laser was reduced to ±0.26% in 2 h when the laser was working in the controlled phase, which was obviously lower than that of ±0.59% when the laser was working in the free-running phase. Simultaneously, the frequency drift of the fundamental-wave 1064 nm laser was suppressed from 21.82 to 9.84 MHz in 1 min when the laser turned to the controlled phase from the free-running phase. The research illustrated that not only the output power stability but also the frequency stability of the fundamental-wave 1064 nm laser could be improved by feedback controlling the intracavity nonlinear loss. The obtained stable single-frequency 1064 nm laser with high output power can be well used in atomic cooling physics, optical parameter oscillators, etc.

## 5. Extension of the Continuous Frequency-Tuning Range

**Figure 16:**
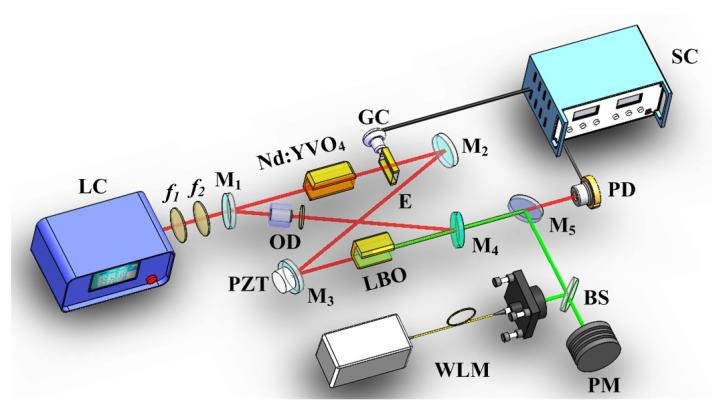
Schematic diagram of super broadband tunable single-frequency CW laser [30]. © [2021] IEEE. Reprinted, with permission, from [30].

**Figure 17:**
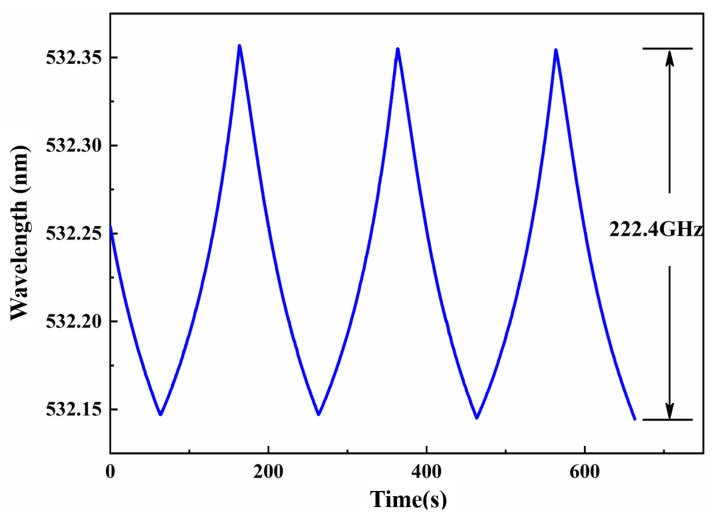
The tuning range of 532 nm single-frequency tunable laser.

An effective method to extend the continuous tuning range of single-frequency CW laser was proposed by combining the intracavity locked etalon with the nonlinear loss [30]. In order to achieve the frequency-tuning of a single-frequency CW laser, the common method was to employ a mode-selection element, such as an intracavity etalon. When the etalon was rotated around its normal axis, or its temperature or the refractive index was changed, the effective optical path of the etalon varied and the frequency-tuning was achieved, but it was not continuous. In order to realize continuous frequency-tuning of the laser, the cavity length of the resonator should be continuously scanned. However, the maximum continuous tuning range was one FSR of the laser resonator, which was about several hundreds MHz. If we wanted to realize the continuous frequency-tuning range of a single-frequency laser beyond one FSR, it was necessary to lock the etalon to the oscillating frequency of the laser resonator. In this case, the maximum frequency-tuning range was still restricted one FSR of the adopted etalon. Because the laser oscillating mode would jump when the laser frequency was tuned to the edge of the FSR of the adopted etalon. Another method for continuous frequency-tuning was to deliberately introduce the nonlinear loss to the laser resonator. Because the nonlinear loss of the lasing mode was half of that of the nonlasing modes, the continuous frequency-tuning range could be up to many times of FSR of the laser resonators. In order to break through the restriction of the FSR of the adopted etalon and realize a wider frequency-tuning range of a laser, the etalon and nonlinear loss were simultaneously introduced to the laser resonator. In this case, the maximum frequency continuous tuning range of the all-solid-state single-frequency CW lasers could be expressed as the formula:

(3) $$\Delta \nu_{\max}=\nu_{FSR}+2\Delta \nu =\nu_{FSR}+\frac{{\left(\frac{\Delta \nu_{H}}{2}\right)}^{2}}{\nu_{FSR}}\times \frac{L}{\eta +L}$$

where ν${}_{\mathrm{FSR}}$ was FSR of the etalon, Δν was unidirectional expanded tuning range, Δν${}_{H}$ was the gain bandwidth of laser gain medium, *L* was linear loss in the laser cavity, and η was nonlinear conversion coefficient. In the experiment as shown in Figure 16, an electro-optic etalon was inserted into the cavity of a single-frequency CW intracavity frequency-doubled Nd:YVO${}_{4}$/LBO laser. The LBO crystal was used as a nonlinear frequency-doubled crystal to convert the 1064 nm laser into a frequency-doubled 532 nm laser. At the same time, the nonlinear loss was introduced. After locking the etalon to the oscillating longitudinal mode of the laser and continuously scanning the cavity length of the laser cavity, a tunable 532 nm laser with a continuous tuning range of 222.4 GHz was achieved in Figure 17. It is expected that a tunable laser whose continuous tuning range covered the whole gain linewidth of gain medium could be realized by optimizing the thickness of etalon and nonlinear loss. Based on the presented method, on the one hand, we have realized a single-frequency laser with the off-limits continuous frequency-tuning range, which can be widely used in high resolution spectral analysis, cold atomic physics, optical frequency standard and other experimental research. On the other hand, a method for realizing a SLM CW laser without any mode hopping and multi-mode oscillation have been achieved.

## 6. Suppression of the Intensity Noise of the High-Power All-Solid-State Single-Frequency CW Laser

### 6.1. Controlling the Nonlinear Loss to Suppress the Intensity Noise of the Fundamental Wave or Second Harmonic Wave

**Figure 18:**
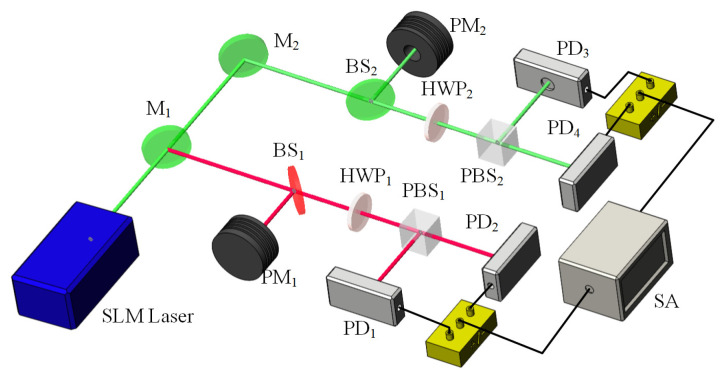
Experimental setup of the noise manipulation [31]. Reprinted with permission from [31] © The Optical Society.

**Figure 19:**
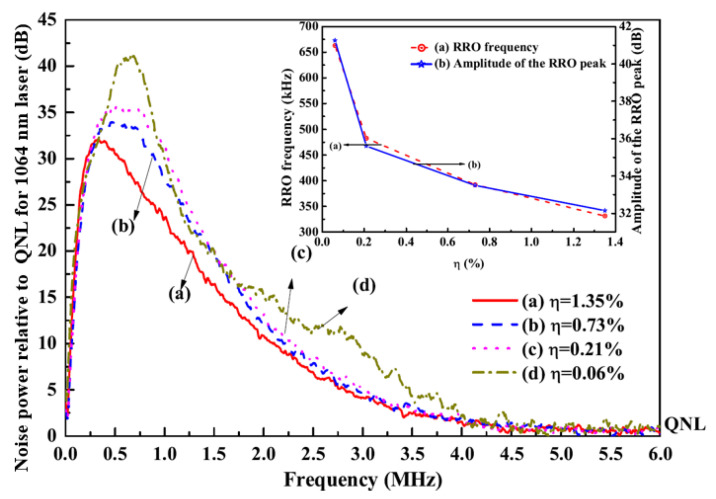
Intensity noise spectrum of 1064 nm laser with different nonlinear conversion coefficients [31]. Reprinted with permission from [31] © The Optical Society.

**Figure 20:**
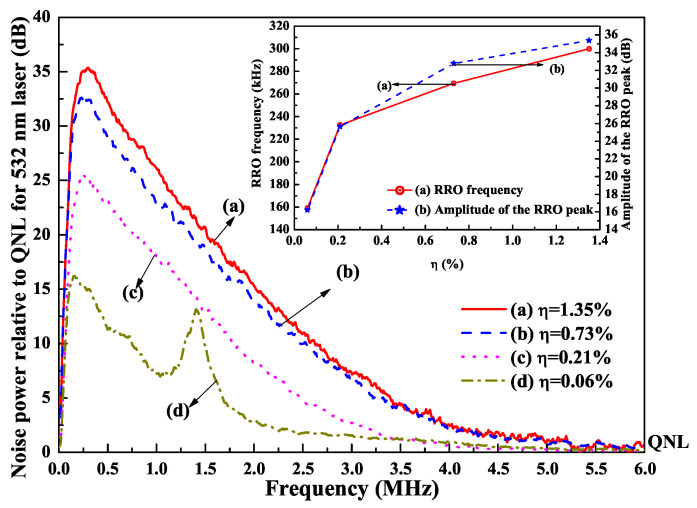
Intensity noise spectrum of 532 nm laser with different nonlinear conversion coefficients [31]. Reprinted with permission from [31] © The Optical Society.

It was also found that the intensity noise of a single-frequency CW laser can be manipulated and suppressed with the assistant of the nonlinear loss. In order to investigate the influence of the nonlinear loss on the intensity noises of the fundamental-wave and second-harmonic-wave lasers, the intensity noise measurement platform was built, which was depicted in Figure 18. The intensity noises of the 1064 nm and 532 nm lasers were separately measured by means of two sets of self-homodyne-detector, each of which included a pair of photodetectors. The photo diodes (PD, ETX500, JDSU corporation and S3399, Hamamatsu corporation) mounted in the photodetectors were used to measure the intensity noise of the 1064 nm and 532 nm lasers, respectively. The optical signals detected by two PDs were amplified by the integrated amplifiers (ADA 4817) and then the amplified photocurrents were combined with a positive/negative power combiner (+/−). The common mode rejection ratio of the used self-homodyne-detector (34 dB) was high enough to satisfy the requirement for measurements. The sum and the subtract photocurrents stood for the intensity noise and the corresponding shot noise limit (SNL), respectively. Finally, the noise spectra of the sum (subtract) photocurrents were analyzed by a spectral analyzer (SA) with the resolution bandwidth (RBW) of 30 kHz and the video bandwidth (VBW) of 30 Hz. In the experiment, the nonlinear conversion rate μ was controlled by detuning the temperature of the LBO crystal. The measured results of the intensity noises for fundamental-wave and second-harmonic-wave lasers were illustrated in Figure 19 and Figure 20, respectively. It was clear that the amplitude and frequency of resonant relaxation oscillation (RRO) peak decreased and shifted to low frequency with the increase of the intracavity nonlinear loss for the fundamental-wave laser. On the contrary, both the RRO peak and frequency of the second-harmonic-wave laser gradually declined and shifted toward low frequencies with the decrease of the nonlinear loss. Comparing the intensity noise spectra of 1064 nm and 532 nm lasers, it had been demonstrated that the intensity noise could be transferred between 1064 nm and 532 nm lasers when the nonlinear loss was introduced into the resonator [31].

### 6.2. Influence of the Pump Scheme on the Intensity Noise of a Single-Frequency CW Lasers

**Figure 21:**
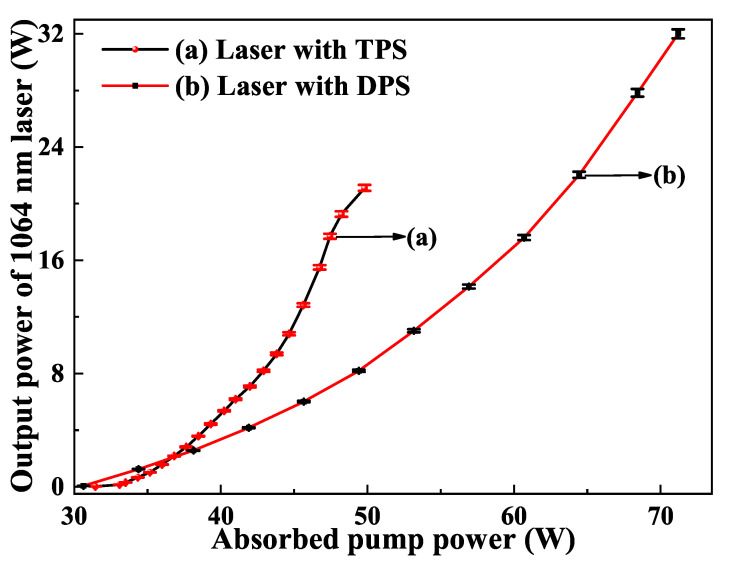
Output power of the 1064 nm laser versus the incident pump power [32]. Reprinted with permission from [32] © The Optical Society.

**Figure 22:**
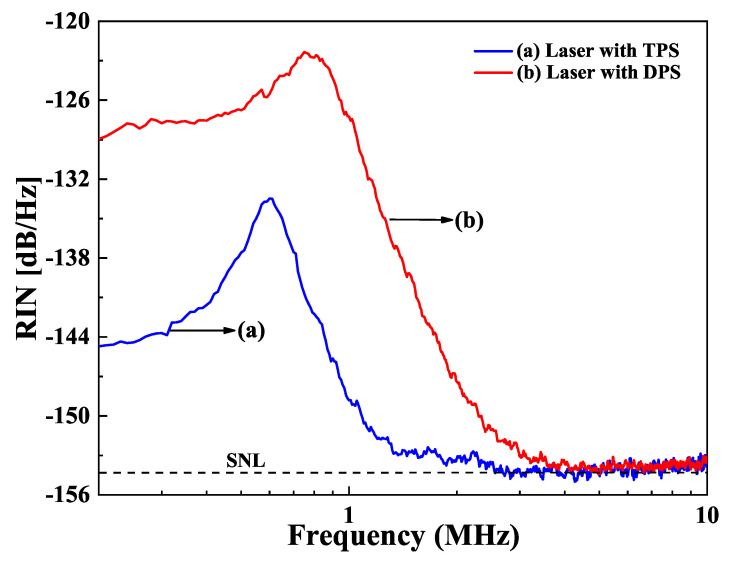
Intensity noise spectra of the single-frequency laser without the nonlinear loss [32]. Reprinted with permission from [32] © The Optical Society.

**Figure 23:**
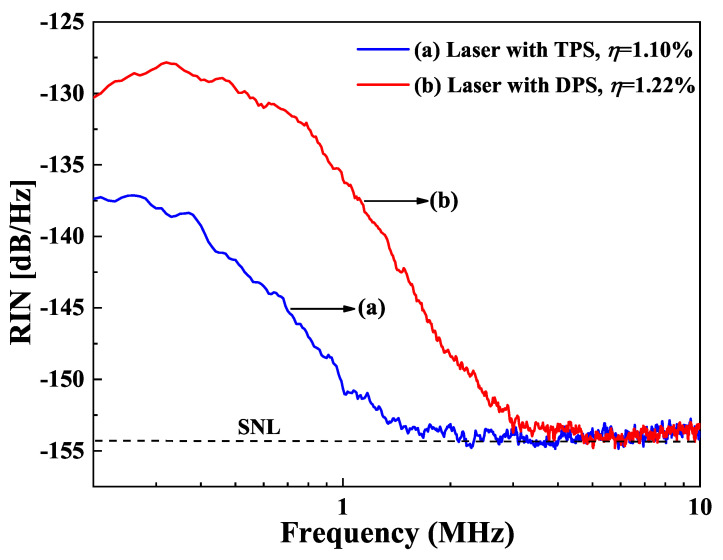
Intensity noise spectra of the single-frequency laser with the nonlinear loss [32]. Reprinted with permission from [32] © The Optical Society.

The influence of the pump scheme on the intensity noise of a single-frequency CW lasers was demonstrated in two single-frequency CW 1064 nm lasers [32]. One was under the 888 nm direct pumping scheme (DPS), and the other was 808 nm traditional pumping scheme (TPS). Because the thermal lens effect of the laser crystal under DPS was less than that of the TPS, the output power of the single-frequency CW laser at 1064 nm was successfully scaled up from 21.1 W to 32.0 W after the TPS was replaced by the DPS in the experiment, as shown in Figure 21. However, the increase of the output power for the single-frequency laser with DPS was accompanied by the elevation of the intensity noise. The measured intensity noise spectra of both single-frequency lasers were shown in Figure 22. It was found that the frequency, the amplitude of RRO peak as well as the SNL cutoff frequency increased from 606 kHz, 20.4 dB/Hz, and 2.4 MHz to 809 kHz, 31.6 dB/Hz, and 4.2 MHz, respectively after replacing the TPS with the DPS. After investigation, it was found that the high intensity noise of a single-frequency laser with DPS resulted from the high dipole fluctuation. For DPS, in order to improve the absorption efficiency of the pump light to achieve high output power, the long laser crystal with the high doping concentration was used in the experiment, which could increase stimulated radiation rate and further enhance the dipole coupling between the large amount atoms and the laser cavity. On this basis, with the assistant of the nonlinear loss, the RRO peak for both TPS and DPS lasers were easy to be suppressed, as shown in Figure 23. The research revealed that the DPS could be employed to scale up the output power of a single-frequency CW laser, while TPS combined with a controlled laser crystal doping concentration could be used to achieve a single-frequency laser with a low-intensity noise. It is believed that this technology can supply a good reference for designing a single-frequency CW laser with good performance to meet different application requirements.

### 6.3. Suppression of the Intensity Noise by Controlling the SER

From above section, the intensity noise of a single-frequency laser can also be suppressed by manipulating the SER. It was well known that the intensity noise of a single-frequency fundamental-wave laser rooted from five noise sources including the vacuum noise caused by the output coupler, the noise coming from the pump source, the noise coming from spontaneous emission, the noise caused by dipole fluctuations, and the noise induced by the intracavity losses. The RRO was mainly stimulated by the vacuum noise coupled from the output coupler, the dipole fluctuations, and the noise caused by the intracavity losses. In the low frequency range below 100 kHz, the intensity noise of the laser was dominated by the intensity noise of the pump source. In the frequency ranges above RRO, the vacuum noise, dipole fluctuations and cavity loss were also the main effects for the intensity noise of the laser. Simultaneously, the spontaneous emission noise had a small effect on the intensity noise of the laser in the whole frequency range, and can generally be ignored. Therefore, the intensity noise spectrum of a single-frequency laser could be given by [33]

(4) $$\begin{array}{cc} V_{f}= & k_{1}\left(\omega_{f},\gamma_{f}\right)V_{\mathrm{vac}}+k_{2}\left(\omega_{f},\gamma_{f}\right)V_{p}+k_{3}\left(\omega_{f},\gamma_{f}\right)V_{\mathrm{spont}}\\ \; & +k_{4}\left(\omega_{f},\gamma_{f}\right)V_{\mathrm{dipole}}+k_{5}\left(\omega_{f},\gamma_{f}\right)V_{\mathrm{losses}\;} \end{array}$$

where *k*${}_{1}$, *k*${}_{2}$, *k*${}_{3}$, *k*${}_{4}$, and *k*${}_{5}$ were the coefficients with respect to the vacuum noise *V*${}_{vac}$ caused by the output coupler; *V*${}_{p}$, the noise coming from the pump source; *V*${}_{spont}$, the noise coming from spontaneous emission; *V*${}_{dipole}$, the noise caused by dipole fluctuations; and *V*${}_{losses}$, the noise induced by the intracavity losses, respectively. And the RRO frequency ω${}_{f}$ was expressed by

(5) $$\omega_{f}=\sqrt{2\kappa G\alpha^{2}}$$

where 2κ was the total photon decay rate, which was expressed by 2κ = 2κ${}_{m}$ + 2κ${}_{l}$. 2κ${}_{m}$ = *Tc*/*L*, and 2κ${}_{l}$ = *δc*/*L* were the photon decay rates induced by the output losses and the intracavity losses, respectively, where *T* was the transmission of the output coupling mirror, δ was the intracavity linear loss, and *c* was the velocity of light. And the damping rate γ${}_{f}$ of the oscillation for *V*${}_{f}$ was expressed by

(6) $$\gamma_{f}=G\alpha^{2}+\Gamma +\gamma_{t}$$

where α${}^{2}$ expressed the intracavity photon number. Γ was the pump rate, and γ${}_{t}$ was the atomic spontaneous emission rate from upper level to lower level. When the pump rate of the laser Γ was given, ω${}_{f}$ and γ${}_{f}$ depended only on the SER *G* of the laser. According to Reference [34], *G* equaled

(7) $$G=\frac{\sigma_{s}\rho l}{nL}$$

**Figure 24:**
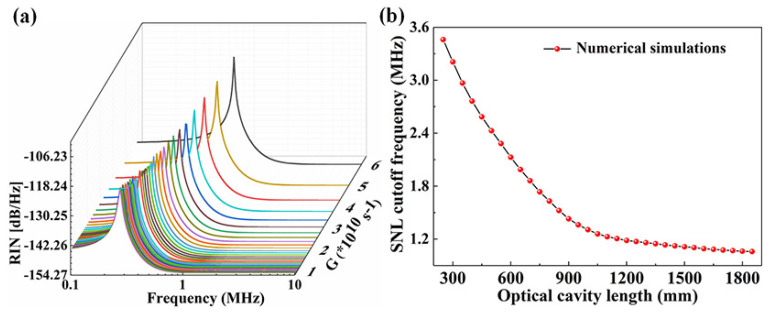
(**a**) Numerical simulations of the intensity noise spectra of the laser with different SER *G*. (**b**) Theoretical prediction of the SNL cutoff frequency versus length *L* of resonator [35]. Reprinted with permission from [35] © The Optical Society.

**Figure 25:**
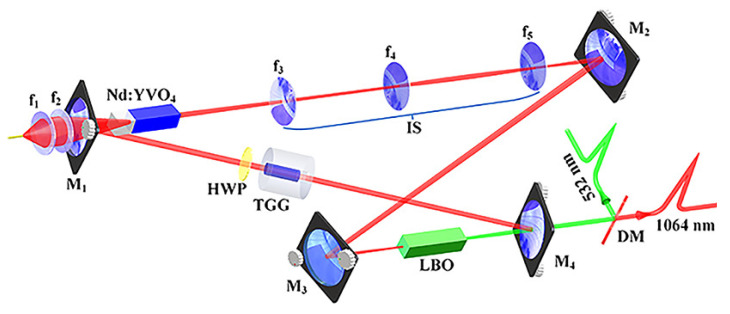
Schematic of the experimental setup [35]. Reprinted with permission from [35] © The Optical Society.

**Figure 26:**
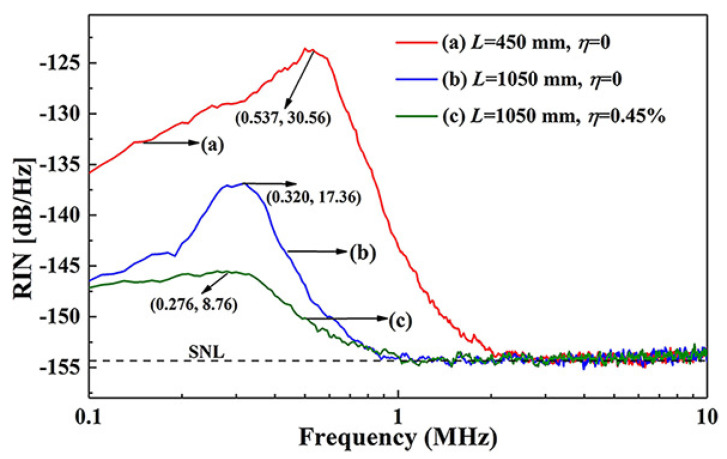
(**a**) Measured intensity noise spectra for L${}_{1}$ laser with η = 0, (**b**) L${}_{2}$ laser with η = 0, and (**c**) L${}_{2}$ laser with η = 0.45% [35]. Reprinted with permission from [35] © The Optical Society.

It can be seen that the SER *G* is proportional to the stimulated emission cross section σ${}_{s}$, atomic density ρ, and laser crystal length *l*, and is inversely proportional to refractive index *n* of the laser crystal and the length *L* of the resonator. The relationship between the SER and the intensity noise spectrum of the laser was simulated first and the results were depicted in Figure 24. The RRO peak of the laser moved toward the low frequency direction, and the amplitude of the RRO peak reduced gradually along with the decrease of SER *G* [35]. Simultaneously, the SNL cutoff frequency also shifted to the low frequency direction when SER *G* decreased. From Equation (7), it can also be found that for a given laser medium, *G* can be simply manipulated by changing *L*. In the experiment as shown in Figure 25, in order to stretch the length of the laser resonator, an optimized imaging system consisting of three lenses was employed and placed in the resonator to control the working stable range of the laser and achieve optimal mode-matching. At the same time, a nonlinear LBO crystal was still adopted to introduce the nonlinear loss, which could further suppress the RRO peak of the laser. The experimental results showed in Figure 26. The frequency and amplitude of the RRO peak were significantly reduced from 537 kHz to 320 kHz and from 30.56 dB/Hz to 17.36 dB/Hz above the SNL when the resonator length of the laser was stretched from 450 mm to 1050 mm, respectively. In particular, the lowest SNL cutoff frequency of the laser reduced from 2.6 MHz to 1.0 MHz. The RRO peak was further suppressed by the nonlinear loss caused by the nonlinear crystal and its amplitude reduced to 8.76 dB. The experimental results agreed well with theoretical predictions. It is clear that the intensity noise of a single-frequency laser can be effectively suppressed by combining manipulation of the SER of a laser with introduction of the intracavity nonlinear loss. The presented intensity noise suppression can ascribe to the low SER in the laser with long cavity length which effectively weakens the coupling strength of all noise sources in the laser resonator. It is believed that the proposed method for reducing intensity noise of lasers will provide a useful technical reference for the design of a laser with low-intensity noise.

## 7. Conclusions

With the developments of sciences and technologies, high-power all-solid-state single-frequency CW lasers have become increasingly important because of their intrinsic advantages including high power, high stability, perfect beam quality and low-intensity noise. In order to improve the whole performance of the high-power all-solid-state single-frequency CW lasers, actively introducing a nonlinear loss to the laser resonator and controlling the SER have acted as both important roles. Based on the nonlinear loss, the presented physical conditions of SLM operation for a single-frequency laser can pave a good way to develop other novel single-frequency lasers. So far, many kinds of single-frequency lasers with the assistant of the nonlinear loss have been demonstrated. For instance, Yang et al. [36] reported an 8 W diamond Raman CW laser that is continuously tunable across the range from 590 nm to 625 nm. Besides that, Bereczki et al. [37] presented a Nd:YAG rod single-frequency ring laser in a dynamically stable resonator providing 55.6 W of continuous, linearly polarized, TEM${}_{00}$ output. Depending on the nonlinear loss, the intracavity linear loss was precisely measured by recording the powers of the fundamental-wave and second-harmonic-wave lasers. Because it was not required to replace any devices of the laser resonator for the proposed method, the high precision and simplicity attracted the favor of the community. After the intracavity linear loss was precisely measured, an optimized laser resonator where both laser crystals and an imaging system were simultaneously inserted was designed and built. As a result, the output power of the single-frequency CW 1064 nm laser was successfully scaled up to 101 W. By studying thermal lens effect of the intracavity magnetic-optical TGG crystal, the passive and active methods were developed to scale up the output power of the single-frequency and frequency-doubled 532 nm laser. In particular, after the DKDP crystal with negative thermal-optical coefficient was employed to compensate for the positive thermal lens effect of the TGG crystal, the output power of the single-frequency 532 nm laser was scaled up to 30.2 W. With the assistant of the nonlinear loss, the power and frequency stabilities and the tuning range as well as the intensity noise of the single-frequency laser can drastically be improved. When the SER *G* manipulation was combined to the nonlinear loss, the intensity noise of the single-frequency CW laser can further improve. Depending on the adoption of the above technologies, various kinds of high-power all-solid-state single-frequency CW lasers have been achieved and applied to lots of fields including quantum technologies, atomic physics and so on.

## Data Availability

Not applicable.
